# Considering Nature and Nurture in the Etiology and Prevention of Picky Eating: A Narrative Review

**DOI:** 10.3390/nu12113409

**Published:** 2020-11-06

**Authors:** Meera D. Patel, Sharon M. Donovan, Soo-Yeun Lee

**Affiliations:** 1 Department of Food Science and Human Nutrition, University of Illinois at Urbana-Champaign, Champaign, IL 61820, USA; meeradp2@illinois.edu; 2 Department of Food Science and Human Nutrition and Division of Nutritional Sciences, University of Illinois at Urbana-Champaign, Champaign, IL 61820, USA; sdonovan@illinois.edu

**Keywords:** picky eating, vegetable intake, eating behavior, exposure, taste sensitivity

## Abstract

Children are often categorized as picky eaters by parents and caregivers for their rejection of foods, such as vegetables, and for exhibiting other difficult mealtime behaviors. However, there are several factors that contribute to these mealtime behaviors, including early feeding practices (i.e., breastfeeding, introduction to solid food), repeated exposure to novel foods, and genetic taste sensitivity to certain compounds. Using the online database of PubMed, a review of the literature on the development of picky eating in children, its outcomes, and intervention strategies was conducted. This review groups the developmental contributors to picky eating into the categories of *nature* and *nurture* and explores the interaction between the two. This paper will also summarize the potential outcomes of picky eating and the various strategies that are currently recommended to mitigate picky eating in young children. However, there is a lack of longitudinal work targeting consistent picky eating behaviors that have the potential to impact long-term food preferences and dietary variety. Future intervention strategies should address the factors that influence the development of picky eating on an individual level.

## 1. Introduction

Picky eating is a commonly used term to describe the mealtime behaviors and food preferences of young children. These behaviors can have long-term consequences related to nutrition and health and should be addressed early in life. Several factors can contribute to the development of picky eating in children; therefore, it is important to understand these elements in order to design appropriate intervention strategies to successfully mitigate picky eating. This paper will illustrate these factors using the categories of *nature* and *nurture* to understand picky eating on an individual level.

### 1.1. Defining Picky Eating

The phenomenon of picky eating is complex and encompasses a variety of child and caregiver behaviors. Currently, there is no widely agreed-upon definition of this phenomenon as the characterization is largely based on parental or caregiver perception of mealtime behaviors. Measuring picky eating is also inconsistent as various tools are used to evaluate these behaviors [1]. However, common behaviors that are associated with picky eating include avoiding the intake of certain food groups, avoiding certain foods based on their sensory characteristics, requiring specific food presentations or preparations, eating a limited variety of food, and not eating an adequate amount of food [1]. Food neophobia, or the unwillingness to try new foods, is another common behavior that is often associated with children who are perceived as picky eaters [2,3]. Food neophobia is considered an evolutionary adaptation to protect one’s self from harm since unfamiliar foods have the potential to lead to illness or death [4]. Although picky eating and food neophobia are often seen as normal childhood behaviors and generally resolve with little intervention, strong and persistent food refusals and very limited ranges of food intake can cause worry amongst caregivers and should be addressed [5,6]. Therefore, it is important to better understand the contributors to picky eating in order to develop effective interventions, as persistent picky eating can have detrimental effects on long-term dietary intake and mealtime behaviors.

### 1.2. Dietary Intake in Picky Eater Children

Children’s early experiences with food are crucial to the development of long-lasting preferences and eating habits. It is suggested that preferences that develop during this time are likely to persist into adolescence and can predict diet quality in adulthood [7,8]. It is widely accepted that a diet rich in fruits and vegetables is important in reducing the risk of obesity and related chronic diseases, as well as heart disease and cancer later in life [7,9]. However, most young children in the United States fail to consume the recommended five servings of fruits and vegetables per day [9]. In fact, 25–30% of young children do not consume even one serving per day. Additionally, the types of vegetables consumed by pre-school aged children in the U.S. often exclude dark-green vegetables, which are rich in micronutrients. Children also tend to prefer highly palatable, energy-dense foods such as sweet, fatty, and salty foods over vegetables [7].

Limited intake of certain food groups and inadequate food intake in children who are perceived as picky may lead to more serious health consequences, such as nutrient deficiencies and growth impediments [2]. Reduced consumption of fruits and vegetables, whole grains, fish and seafood, meat, and unsweetened cereals have been noted in picky eaters when compared to non-picky eaters. A difference in the intake of certain micronutrients, including zinc and iron, between the two groups has been observed as a result [10]. Within the Avon Longitudinal Study of Parents and Children (ALSPAC) in the U.K., picky eater children were identified from questionnaires completed by the parent when the child was 2, 3, 4.5 and 5.5 years of age. Dietary intake was evaluated using 3-day food records collected from the same children at 3.5 and 7.5 years of age. Dietary intake at 3.5 years of age was compared between children who were identified as either a picky or non-picky eater at 3 years of age. The 7.5-year assessment compared dietary intake longitudinally in children defined as persistent picky eaters from 2 to 5.5 years of age. Results indicated that picky eaters consumed slightly less protein than non-picky eaters, less carotene, and significantly less iron and zinc than non-picky eaters. Picky eaters were also more likely to have iron and zinc intakes below the U.K. reference nutrient intake (RNI) levels for these nutrients than non-picky eaters. The authors attributed these differences to lower intakes of meat, fish, vegetables, and fruits in picky eaters. Additionally, picky eaters consumed more sugary foods and drinks than non-picky eaters at 7.5 years of age [10]. A recent review examining the relationship of childhood picky eating with food and/or nutrient intakes and growth, found that picky eaters consumed significantly lower amounts of micronutrients including iron, zinc, vitamins A, B6, C, and E, thiamine, riboflavin, and niacin compared to non-picky eaters in at least one of the included studies. The association of picky eating with body weight and growth was inconsistent based on the included work, however, and warrants further research [2]. A longitudinal study conducted in Quebec on a cohort of pre-school aged children found an association between body weight and eating behaviors. Child eating behavior questionnaires were completed by the parent when the child was 2.5, 3.5, and 4.5 years of age. Body mass index (BMI) was measured at 4.5 years of age. Mean BMI of the children that had been reported as a picky eater at one or more timepoint was significantly lower than that of children that had never been reported as picky eaters [11]. A study by Ekstein et al. [12], which included a non-picky eater control group, found a significant association between perceived picky eating behavior and being underweight [12]. On the other hand, work by Wright et al. that followed a group of children from birth to 30 months old, concluded that children described as picky were only slightly shorter and lighter than their non-picky counterparts, and that these differences were not significant [13]. In another study following 71 children from 42 to 84 months of age, no significant differences were observed in mean height and weight based on picky eater status [14]. Due to the potential detrimental impact of picky eating on these outcomes, it is an important consideration when emphasizing nutritionally balanced diets in young children.

Some elements of picky eating behavior may be attributable to innate, genetically-determined taste preferences present in young children [7]. We propose that the phenotype of picky eating represents an interplay between genetics (*nature*) and environment (*nurture*), as repeated exposure to less palatable foods can lead to greater acceptance [4]. This review summarizes the evidence for *nature* and *nurture* as developmental contributors to picky eating and explores the interaction between the two. Potential outcomes of picky eating and the various strategies that are currently recommended to mitigate picky eating in young children are also discussed. While previous reviews on this topic have attempted to provide an overview of studies that examine a specific factor or outcome in relation to an eating behavior, this review aims to bridge the gap between factors arising from both *nature* and *nurture* and the perception of picky eating.

## 2. Materials and Methods

A review of the literature was conducted in PubMed using ‘picky eating’, ‘vegetable intake’, ‘taste genetics’, and ‘child food preferences’ as keywords. Papers were also found within reference lists of previous reviews on related topics and in other eminent papers in the field. As this is a narrative review, no specific inclusion or exclusion criteria were outlined except that all included articles were in English. A large portion of the included research was conducted on pre-school aged children or younger; however, some of the referenced review articles included findings from a wider range of ages. The majority of the included studies were cross-sectional in nature with a few being follow-up studies.

## 3. Development of Picky Eating

**Figure 1:**
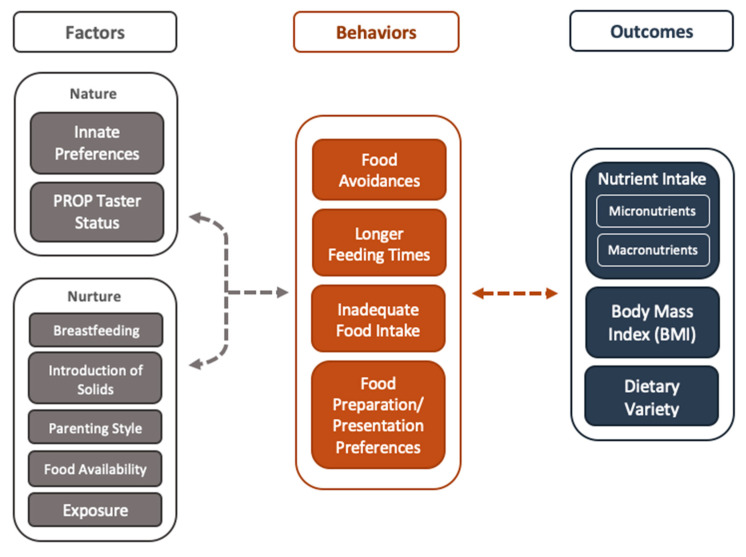
Concept flow diagram illustrating genetically determined (nature) and environmental (nurture) factors affecting child picky eating behaviors as observed by caregivers and their developmental outcomes.

As illustrated in Figure 1, picky eating is characterized by a variety of mealtime behaviors and has been associated with biological (*nature*) and environmental (*nurture*) factors. As noted above, these mealtime behaviors have the potential to influence health and nutritional outcomes in children. It is important to note that Figure 1 does not present an exhaustive view of the factors associated with picky eating, rather, it outlines the factors, behaviors, and outcomes that will be discussed in this review. There are other outcomes such as gastrointestinal issues and social outcomes that may result from picky eating in children; however, they are not discussed in this review in order to provide a focused discussion. This section will explore factors within the categories of *nature* and *nurture* as well as the interaction between the two. Considering picky eating as multifaceted in this way will better inform the mitigation of its various behaviors and outcomes.

### 3.1. Nature

#### 3.1.1. Taste Perception

##### Bitter Taste

The ability to taste certain bitter thiourea compounds, such as 6-n-propylthiouracil (PROP) and phenylthiocarbamide (PTC), is genetically determined. In the U.S., about 70% of the Caucasian population can detect the taste of PROP and are called tasters, while 30% are insensitive to the taste of PROP and are called non-tasters [15]. This distribution varies by geographic location and/or ethnicity [16]. PROP/PTC tasters have shown to be more sensitive to bitter compounds such as caffeine and quinine as well as common food additives, such as saccharin, potassium chloride, sodium benzoate, and potassium benzoate, which have bitter aftertastes. PROP tasters also have more food dislikes overall. In addition to bitterness, PROP taster status may be related to perception and preference for sweet taste, fat texture, and the oral sensation from capsaicin, the active irritant compound in chili pepper [16].

Studies suggest that genetic taste factors such as PROP sensitivity may be informative for understanding changes in food preferences and dietary choices over time [15]. For example, the relationship between PROP taster phenotype and vegetable acceptance and intake in pre-school aged children was explored by Bell & Tepper [17]. In this free-choice intake test, PROP non-taster children consumed more vegetables and rated raw broccoli higher than did PROP taster children during a hedonic test, suggesting a relationship between PROP taster status and vegetable acceptance [17]. Mennella et al. [18] provided an overview of studies that further explored the association between the genetics of taste receptors and taste perception in pediatric populations. These types of studies have been conducted mainly in children aged 3 to 14 years of age, but the effect of PROP taster genetics on taste perception has yet to be explored in infants [18].

A study conducted by Cole et al. [19] in pre-school aged children determined that genetic variation in certain bitter taste receptor genes was associated with picky eating behaviors such as reduced dietary variety and struggle for control at mealtime. Children with limited dietary variety in this study had lower BMI z-scores than non-picky eaters [19]. In a 6-year follow-up study conducted by Oftedal & Tepper [20], the interaction between PROP taster status, gender, and eating attitudes, had a modest, but not statistically significant, relationship with weight status in children. While not significant, trends in the relationship between BMI percentile and PROP taster status were noted at the time the study was conducted. In boys, PROP taste sensitivity was inversely related to BMI percentile, whereas, in girls, the opposite trend was observed. PROP non-taster girls also showed a decrease in BMI percentile from baseline (preschool-age) to follow-up (~10 years of age). The authors consider that the influence of PROP on weight during childhood is variable over time and across genders given differences in developmental rates, leading to this inconsistency in findings [20].

##### Sweet Taste

The preference for and ability to detect sweet taste are innate, evolutionarily-driven attributes in children since sweet taste typically indicates caloric density, which is important during periods of growth [18,21]. This is supported by research showing that higher levels of sweet taste are preferred during childhood and decline to adult levels during mid- to late-adolescence as adulthood coincides with the termination of growth [21]. The sweetness concentrations preferred by children in studies have shown to relate significantly to the levels of sweetness that they prefer in their cereals and beverages [22]. This suggests that children are likely to overconsume these sugary, energy-dense foods [18].

##### Fat Sensitivity

Along with a sensitivity to certain bitter tastes, PROP tasters are more sensitive to sweet tastes and the oral sensation of fattiness [15]. In fact, it has been shown that PROP tasters were able to discriminate between high- and low-fat salad dressings better than non-tasters. Although tasters did not have a preference between the two, non-tasters preferred the high-fat sample [16]. Keller et al. [15] aimed to understand the relationship between PROP taster status and bitter and fatty properties in pre-school children, aged 4 to 5 years. Results indicated significantly lower acceptance ratings for raw broccoli and American cheese in PROP tasters versus non-tasters. Raw broccoli contains bitter-tasting isothiocyanates, which are released during cooking, and American cheese contains bitter-tasting calcium chloride to which PROP taster children may be more sensitive. Additional findings from this study included PROP non-tasters consuming more servings of discretionary fats than PROP tasters and a gender effect of taster girls having the lowest acceptance ratings of full-fat milk [15].

##### Changes in Taste Perception with Age

Age also heavily influences taste perceptions. As noted above, children tend to prefer higher concentrations of sweetener than do adults [23] and may also perceive bitter taste differently, partly, as a result of genetics [18]. However, children displayed greater sensitivity to bitter taste than their mothers of the same genotype, demonstrating that genetics alone does not explain higher bitter sensitivity in children. In addition, these children were perceived as more emotional by their mothers than children without bitter sensitivity [22]. Innate and genetic taste sensitivities play a key role in the development of picky eating behaviors in young children but are not the only factors involved. A later section will introduce factors categorized as *nurture* elements that also impact picky eating.

#### 3.1.2. Genetics

It has been shown that traits such as food neophobia and ‘food fussiness’ are highly inheritable [24,25]. A study conducted by Fildes et al. evaluated pairs of three-year old twins to determine if common genetic influences underlie the association between food fussiness and fruit and vegetable liking. Food liking measures and the Child Eating Behavior Questionnaire (CEBQ) were completed by parents when the twins were 3.5 years of age. Genetic modeling was used to estimate common genetic influences of food fussiness and fruit and vegetable liking. Results indicated that food fussiness and fruit and vegetable liking share common genetic factors [24]. A study done with 8–11-year-old twins investigated the influence of genes and environmental factors on variation in food neophobia. Genetic differences explained 78% of the variation in food neophobia scores, while 22% was explained by environmental factors specific to the individual. The authors concluded that, in addition to genetics, individual environmental influences are important to consider when addressing food neophobia in children [25]. Furthermore, a systematic review conducted by Diószegi et al. synthesized the findings of studies investigating genetic polymorphisms that contribute to variability in taste preferences. Significant associations between certain gene variants and preferences of bitter and sweet taste as well as responsiveness to fat were reported [26]. A recent study conducted in children with obesity investigated the effect of lipid sensor gene alteration on bitter taste and fat perception as well as on preference of palatable foods. The lipid sensor genes were significantly more methylated in children with obesity than in lean children. Children with obesity exhibited a higher detection threshold for bitter taste and for fatty acid perception. A positive association was observed between the methylation of these genes and the intake of calories from highly palatable foods [27].

### 3.2. Nurture

#### 3.2.1. Early Life Exposures

##### In Utero

The impact that experiences with flavor during early life have on food preferences and intake later in life has been exhibited in several studies. In the womb, fetuses are exposed to and swallow the fluid of the amniotic sac [28]. Amniotic fluid flavor is highly variable and directly reflects the foods present in the mother’s diet. Preferences for the flavors present in amniotic fluid are therefore heightened following birth [29]. It has been shown that in the days after birth, infants orient toward the odors in their amniotic fluid that contained flavor compounds consumed by their mothers while pregnant. The permeation of flavors such as garlic and anise in the amnion influences the infant’s facial and mouthing responses to those flavors [28]. In a study in which mothers were randomly assigned to consume carrot juice during the last trimester of their pregnancies, infants (at about 5.7 months old) were rated by their mothers as having enjoyed carrot-flavored cereals more than those whose mothers did not consume carrot juice. Additionally, the infants that were exposed to carrot flavor during pregnancy consumed more carrot-flavored cereal than plain cereal compared to infants that were not exposed to carrot flavor (*p* = 0.34) [28,29]. A recent review by Spahn et al. [30] provides an overview of studies that investigated the influence of maternal diet during pregnancy and lactation on flavor transfer to the amnion and breastmilk.

##### Breastfeeding

Influences on flavor preferences continue outside of the womb via early feeding practices. The first several months of life are an important period during which flavor learning takes place [29]. In the aforementioned carrot juice study, a group of breastfeeding mothers was assigned to consume carrot juice during the first three months postpartum. Their infants had enhanced responses to carrot flavor when introduced at weaning compared to those that were not exposed [28]. Earlier work by Mennella and Beauchamp indicated that garlic and alcohol consumption by mothers impacted breast milk flavor and the suckling behavior of their infants [31,32,33,34]. Mennella and Beauchamp also conducted a study investigating the effects of vanilla flavor in breastmilk on infant feeding responses. Infants fed for 25% longer and consumed 20% more milk from the breast when the milk was flavored with vanilla [35]. This suggests that flavors in the mother’s diet that are transmitted via breastmilk are detectable by infants [30]. Breastfeeding can also help to facilitate the acceptance of novel foods, fruits and vegetables, and solid foods [36,37].

##### Formula Feeding

The flavors in infant formula can vary greatly based on processing and composition. For example, hydrolyzed casein formulas (HCFs) are more bitter, sour, and savory in flavor than breastmilk and bovine-milk formulas (MFs). This difference in flavor is, in part, a result of the high free amino acid content of HCFs. Mennella et al. [38] investigated the effects of these different kinds of milk and formulas on acceptance of sweet, sour, bitter, salty, savory, and plain cereals in four-to nine month old infants. The HCF-fed infants ate more of the savory, sour, and bitter cereals than did breast-fed or MF-fed infants. They also exhibited fewer negative facial expressions in response to the savory and bitter cereals during feeding [38].

Another study used extensively hydrolyzed protein hydrolysate formula (ePHF) to understand the effects of exposure to its distinct flavor on acceptance of broth with and without the amino acid glutamate, which imparts a savory flavor. Infants fed ePHF for three or eight months ate more of the savory, glutamate-containing broth than plain broth compared to the control group that was fed MF. The length of time that infants received ePHF was also significant as the group that was fed ePHF for just one month did not differ from the control group [39].

There is also evidence that indicates long-term effects of these early life experiences with flavor. The effects of three types of commercially available infant formula were evaluated in four-to-five year old children that received either MF, soy formula (SF), or HCF as infants. Children fed SF, which is described as being more sour and bitter than MF, preferred bitter-flavored apple juice and were significantly more likely to prefer broccoli than children fed MF. Those fed HCFs were more likely to prefer sour-flavored juice compared to MF-fed children [40]. These findings support the idea that early flavor experiences can help shape food preferences.

##### Introduction to Solid Foods

As reviewed by Blissett et al., when and what foods are introduced during weaning shapes the development of food acceptance and behaviors [41]. The age at which children are introduced to complementary foods may play a role in their readiness to consume new foods. Those who receive solids after ten months of age have lower dietary variety, consume fewer fruits and vegetables, and exhibit more feeding problems [42,43]. Exposure to a variety of flavors during the so-called “sensitive period” for introduction to solids, between four to five months of age, is proposed to promote food acceptance later in life [41]. The American Academy of Pediatrics (AAP) recommends exclusive breastfeeding for around six months, with the possibility of introducing solids at four months. It also recommends introduction of potential allergens before six months to prevent the development of allergies [44]. Additionally, children who receive a variety of fruits and vegetables early in weaning more readily accept new foods during weaning [41]. As there is still relatively little evidence on the impact of age of introduction to solids on picky eating, further research in this area is warranted.

#### 3.2.2. Parenting Style

The designation of a child as a picky eater by a parent or caregiver is often based on various observed behaviors, as mentioned earlier. These can include inadequate food intake, avoiding the intake of certain food groups or sensory categories, requiring food to be prepared in a specific way, refusal of new or familiar foods, and eating a limited variety of food [1,45]. Research conducted by Podlesak et al. [45] suggested that parenting style is related to mealtime behaviors exhibited by both the parent and the child, and to the perception of picky eating and non-picky eating behaviors by parents. Using the measures of demandingness (control) and responsiveness (warmth), Baumrind described three core parenting styles, which are summarized in Table 1 [46]. In the study conducted by Podlesak et al. [45], parents of two-to-five year old children responded to two online surveys, the Mealtime Assessment Survey (MAS) [47] and the Parenting Styles and Dimensions Questionnaire (PSDQ) [48]. The MAS measured the frequency of specific parent and child behaviors at mealtimes, while the PSDQ measured parenting style. Authoritative parenting was positively associated with non-picky eating behaviors and strategies that promoted positive eating habits in children, whereas authoritarian and permissive parenting styles were positively correlated with negative mealtime strategies and the perception of picky eating behavior. Thus, this study concluded that implementing authoritative parenting strategies such as encouragement, modeling, and child involvement could help combat picky eating behaviors [45].

#### 3.2.3. Parental Feeding Practices

Strategies used at mealtimes are often influenced by parenting style [45]. These parental feeding practices can also impact children’s fruit and vegetable acceptance and intake [7]. Feeding practices can vary by strategy, emotional tone, and effectiveness. Practices that are responsive to child behaviors and use negotiation rather than pressure are associated with lower neophobia and higher motivation to try vegetables. They are typically child-centered practices and can include encouragement, praise, parent-modeling of vegetable intake, use of mealtime rules, and monitoring of low-nutrient foods. The use of non-food rewards has also been shown to increase the inclination to try vegetables. On the other hand, practices that are unresponsive and permissive are related to low vegetable acceptance and intake. This can include allowing children to decide which foods are offered at mealtimes, using desirable foods as a reward for trying undesirable foods, indulging children’s demands, and applying pressure to eat [7]. A systematic review by Cole et al. cites several studies indicating that negative, non-responsive feeding styles are positively associated with picky eating [49]. Positive feeding styles require a high level of attention and cognitive input on the part of the parent as well as confidence that the child will accept foods if time and effort is put in [7]. Table 1 includes findings from studies that investigated the relationship between parenting style and parental feeding practices. Across these studies, the authoritative style was associated with food modeling and parental monitoring or structuring of mealtime [50,51]. Authoritative parenting was also positively associated with vegetable availability and consumption compared to authoritarian parenting. This suggests that implementing authoritative feeding practices may be beneficial [52].

#### 3.2.4. Food Availability

The food environment of a household is vital to children’s food behavior development. The opportunity that a child has to consume a food that is not typically available at home is small. Children living in homes with higher availability of fruits and vegetables tend to consume more of these foods. This also applies to unhealthy foods. More access to sweet and salty foods leads to greater consumption of these foods [53]. Exposure to a greater variety of foods at home can also influence a child to try new foods outside of the home [41,54]. In a study conducted by Arcan et al. [55], having just one additional fruit or vegetable available in the home was associated with an 11% higher likelihood that a child would adhere to the USDA’s MyPlate guideline of *filling half a child’s plate with F&V* [55].

The socio-economic status of a family can also dictate the foods that are available in the home, and therefore also plays an indirect role in the development of a child’s food preferences [41,56]. In fact, mothers who reported food insecurity were less likely to have fruit readily available in the home than mothers who reported food security [57]. Socio-economic status can also moderate parental response to children’s eating behaviors. For example, low-income mothers may provide foods that are already liked (often unhealthy options) in an effort to reduce food waste if they know their child will reject a novel, healthier food. Thus, children from food-insecure homes may consume more energy-dense foods than children from food-secure homes as these foods tend to be innately preferred and inexpensive [57]. Therefore, food security and home food availability should be considered in the implementation of interventions to increase fruit and vegetable intake in young children.

#### 3.2.5. Exposure

Several studies indicate that an individual’s experience with and exposure to a food plays a critical role in the preference for that food [4,37,58,59,60]. This highlights the concept of food neophobia and its role in food selection. As the ingestion of a new substance is associated with danger and risk, it is typically avoided. Familiar foods, on the other hand, are considered safer to consume since they are not associated with negative outcomes. Nearly 40 years ago, however, Birch and Marlin demonstrated that experience with a novel food can enhance the liking of that food by increasing its familiarity [4]. Since that time, a plethora of additional work has supported these early observations. A review by Mennella et al. concisely outlines many of these findings [61].

In general, children prefer foods that are more familiar to them. It has been observed that upon being offered a novel food, young children hesitate to taste it, and many will spit it out. However, even low levels of exposure to a novel food can increase its acceptance and preference [4]. Birch et al. also investigated the type of exposure needed to affect food preferences [60]. They studied whether tasting a novel food was necessary or if simple visual exposure to it could have an effect on visual preference and potentially even on taste preference. Since food contains visual, gustatory, olfactory, tactile, kinesthetic, and common chemical stimuli, it offers multiple means of exposure with which to gauge changes in preference. Both visual and taste exposures were tested with varied exposure frequencies of 5, 10, and 15 times within each type. Visual exposure effects were observed in increased visual preference while taste exposure increased taste preference. Additionally, 10 or 15 exposures to a novel food significantly increased liking of the food [60]. Ten exposures to artichoke increased artichoke acceptance in a study conducted by Caton et al. [58]. Many parents do not continue to present a novel food this many times, however. Additionally, just increasing visual exposure by presenting the food to the child is not sufficient for increasing liking for the taste of the food [60]. Age can also influence the outcome of repeated exposure strategies as the process of developing new food preferences can become more difficult with age. A four- or five-year-old child may need as many as 15 exposures of a novel food to increase acceptance, while a two-year-old may only need 10 [62].

A study conducted by Gerrish & Mennella [63] subjected formula-fed infants to a nine-day exposure period, during which they were fed carrots, potatoes, or a variety of vegetables that did not include carrots. Before the exposure period began, the infants were all fed puréed carrots during a testing session. During separate testing sessions following the exposure period, the infants were fed puréed carrots and puréed chicken. The amount of food consumed was determined by weight and enjoyment of the food was rated by the mothers after each session. It was found that repeated exposure to carrots or a variety of vegetables was associated with increased carrot consumption following the exposure period. Exposure to a variety of vegetables also supported the acceptance of puréed chicken following the exposure period. Exposure to a single vegetable, however, did not have the same effects. These results indicate that exposure to a variety of flavors can enhance the acceptance of new vegetables and novel foods from other categories [63]. Additional evidence of this comes from a later study done by Mennella et al. [37]. When subjected to 8 days of exposure to pears or a variety of fruits not including pears between meals, infants (between four and nine months of age) consumed more pears. Eight days of exposure to a variety of vegetables between and within meals also resulted in an increase in the acceptance of carrots, green beans, and spinach [37].

Coulthard et al. [62], investigated how the type and frequency of fruit and vegetable introduction at six months of age would predict fruit and vegetable intake at seven years of age in the ALSPAC cohort [62]. The data from questionnaires filled out by mothers when their children were six months and seven years old were examined. Both surveys included questions about the frequency of consumption of certain foods and drinks. The age of introduction to each food was also asked in the six-month survey. Specifically, mothers were asked to report if their child had consumed fruits and vegetables in ready-prepared form, raw, and/or home-cooked at six months of age. Higher frequency of consumption of home-cooked vegetables at six months was strongly associated with a higher frequency of vegetable consumption at seven years of age (*p* < 0.001). This pattern was also seen in fruit consumption as a higher frequency of fruit consumption at six months was strongly associated with a higher frequency of fruit consumption at seven years of age (*p* < 0.001) [62]. These findings indicate the positive impact of early fruit and vegetable exposure on long-term consumption.

There are several elements in the development of picky eating that can be grouped into the *nurture* category including parental practices, breastfeeding status, food availability, and exposure to novel foods. In the next section, we will describe how multiple factors from both the *nature* and *nurture* categories can be involved in forming mealtime habits during early childhood.

### 3.3. Interaction between Nature and Nurture

Several factors interact to shape each individual child’s eating behaviors and food preferences [41]. Early experiences including flavor exposure in utero and via breastmilk interact with genetic differences in flavor perception in a complex way to establish food preferences [29,30]. Elements of *nurture*, such as exposure to various tastes and parental feeding practices, can alter child feeding behaviors that may be based on inherent taste sensitivity. The food environment can also largely impact food preferences. On the other hand, a child’s innate preferences and behaviors, as well as their temperament, can affect *nurture* components like parenting style and food availability [41]. In their study of fruit and vegetable intake in two to five-year-old children and their mothers, Coulthard and Blissett [9] reported that children who were sensitive to taste/smell stimuli were less likely to model the fruit and vegetable consumption of their mothers. This indicates that the effect of parental modeling of food behavior can be influenced by the child’s sensitivity to taste [9]. Thus, Walton et al. describes picky eating as a bi-directional concept in which parental feeding practices can influence the child’s behavior and vice versa [6]. This is further emphasized in the systematic review conducted by Cole et al., which summarizes the correlates of picky eating behavior and food neophobia in young children. This review outlines the importance of the bi-directional nature of feeding interactions within the child-parent dyad [49]. This knowledge also suggests that factors leading to the development of picky eating behaviors are multidimensional and will vary from child to child. There is limited research on the interaction amongst these various factors in relation to picky eating [64]. Future research and intervention strategies must, therefore, take into account the various combinations of *nature* and *nurture* elements at play in the development of picky eating in children and should be individualized.

## 4. Mitigating Picky Eating

### 4.1. Improving Sensory Characteristics

As discussed earlier, vegetables often possess a bitter taste, due in part to dietary phytonutrients, and lack innately liked sweet taste. This low palatability leads to a low intake of vegetables, such that most Americans across all age distributions do not meet dietary recommendations in this category [65]. As the phytonutrients contained in vegetables impart health benefits, such as antioxidant and anticarcinogenic effects [66], masking their bitter flavors to increase intake rather than removing these compounds is favored.

Studies have investigated using herbs and spices as a method of improving vegetable flavor and preference [66,67,68]. Among adult vegetable likers participating in a study conducted in 2018 by Feng et al. [66], seasoned vegetables were significantly preferred over unseasoned vegetables. The vegetables used in this study included broccoli, cauliflower, green beans, and carrots. The seasonings included soybean oil, table salt, ground ginger, garlic powder, dried ground cayenne chili pepper, onion powder, dried dill weed, ground black pepper, ground coriander seed, and dried parsley. The combination of seasonings used varied for each vegetable since each vegetable contains a different flavor profile. The authors also suggested that optimal pairings of seasonings and vegetables should be determined, since complex interactions among aromatic compounds occur when vegetables are mixed with seasonings, thus impacting sensory properties. This approach may more efficiently increase the acceptance of vegetables [66]. However, as this study was conducted with vegetable likers, similar research should be conducted in those who do not like vegetables. In children, who are the target population of such interventions, Carney et al. [69] explored the effect of adding an herb and spice blend to vegetables on vegetable intake. During one session, children were given three servings of cooked carrots, each with a different spice blend, as part of a test meal that also included macaroni and cheese, applesauce, milk, and water. During a second session, the three carrot servings had the same herb and spice blend. Results indicated that the effect of the herb and spice variety differed based on the PROP taster status of the children. Children who were PROP tasters consumed more carrots than PROP non-tasters when presented with carrots with three different herb and spice blends. The authors of this study concluded that increasing flavor variety using herbs and spices may increase vegetable intake in PROP tasters [69].

Improving upon the palatability of vegetables using dips is another method for increasing vegetable intake. A study of preschoolers looked to determine whether reduced-fat herb dips would increase the children’s liking, willingness to consume, and consumption of disliked or moderately liked vegetables [70]. Serving vegetables with a liked herb dip increased acceptance and consumption of novel or disliked vegetables. More of the disliked vegetable was consumed when served with an herb dip than when it was served alone (*p* < 0.05). The amount of celery eaten increased by 62% and the amount of squash eaten more than doubled when paired with an herb dip. This outcome indicates that caregivers, childcare centers, and schools may be able to increase children’s vegetable acceptance and consumption by using small portions of reduced-fat herb dips [70].

Fisher et al. [71] sought to determine whether a palatable dip combined with repeated exposure would increase liking and intake of a raw vegetable in predominantly Hispanic preschool-aged children with sensitivity to the bitter compound PROP [71]. Broccoli, a moderately-liked vegetable, was offered to the children with four different dip variations: salad dressing as a dip; light dressing as a dip; mixed with dressing; or without dressing. After an exposure period of seven weeks, children’s broccoli liking increased independently of dip variation and bitter sensitivity. Bitter taste-sensitive children, as the majority of the Hispanic children were, ate 80% more broccoli with dressing than when it was served without dressing. However, the results differed between light and regular dressing and PROP taster status; in bitter taste-insensitive children, serving broccoli with dip did not increase broccoli intake. The authors concluded that offering a palatable dip, along with repeated exposure, can promote the intake of a moderately-liked raw vegetable in Hispanic children with bitter sensitivity [71].

Cooking also offers an opportunity for increasing vegetable palatability. For example, during cooking, bitter flavonoids and isothiocyanates present in raw broccoli are released, potentially increasing acceptability upon consumption. In addition to causing changes in flavor, cooking also softens the texture of broccoli making it preferred over raw broccoli as children tend to prefer softer foods [15]. Some studies suggest that children prefer the crunchy texture of raw vegetables, but those that are difficult to chew, and are therefore disliked or avoided, may benefit from the softening that occurs during cooking [72].

### 4.2. Combining Intervention Strategies

A review conducted by Nekitsing et al. aimed to identify successful intervention strategies to increase vegetable intake in children two to five years of age [73]. The majority of studies utilized educational, taste exposure, and stealth intervention strategies as well as strategies that paired two or more foods together. Other strategies included increasing food accessibility, changing portion size, changing the way food is served, rewards, modeling, choice, variety, and visual presentation. Further detail on what these strategies entailed can be found in the original publication [73]. Some studies used multiple strategies to enhance vegetable intake, but it was determined that taste exposure/repeated exposure had a greater effect than other strategies and combinations. The number of taste exposures was also found to be an important factor. In addition, when the vegetable used in the study was initially unfamiliar or disliked, the effect of the intervention was greater than when the vegetable was initially familiar or liked. In both cases, however, the intervention strategies were effective. The authors suggest that strategies should be combined not only to increase the intake of a target vegetable but also to improve the usual consumption of vegetables. Repeated exposure combined with education for children and their parents/caregivers is recommended to simultaneously improve the consumption of a target vegetable and a child’s usual vegetable intake [73]. Holley et al. [74] conducted a study investigating the effectiveness of various intervention strategies for increasing acceptance of a disliked vegetable in pre-school aged children. The study included 115 parent-child dyads assigned to one of four conditions or to a control group. The four intervention conditions were repeated exposure, modeling and repeated exposure, rewards and repeated exposure, and modeling, rewards, and repeated exposure. The disliked vegetable was presented by the parent for fourteen days with liking and intake being measured both pre- and post-intervention. The modeling, rewards, and repeated condition, as well as the rewards and repeated exposure condition, saw significant increases in consumption following the intervention compared to the control group. Significant differences between groups were also observed in post-intervention vegetable liking, with the highest ratings seen in the modeling, rewards, and repeated exposure group [74]. Thus, these combinations of intervention strategies have the potential to improve children’s vegetable acceptance and intake.

In children with heightened sensitivity to PROP, or high levels of food neophobia, repeated exposure alone may not be sufficient to increase vegetable intake. To increase acceptance of bitter vegetables, which have been shown to require more intense conditioning strategies, flavor–flavor learning (a strategy in which un-liked vegetables are paired with a liked flavor) may be more effective [72]. The most promising strategies may even be a combination of repeated exposure and flavor–flavor learning. Additionally, pairing a bitter vegetable with a child-friendly name can help increase its acceptance [72,75]. Packaging vegetables in containers that appeal to children and including stickers as incentives have shown to double vegetable intake in children who initially consumed low amounts. Creative presentations of vegetables in these ways is an area that can be further explored in an effort to increase vegetable intake in children [72].

A study concentrating on a behavioral intervention for parents of children with problematic mealtime behaviors was conducted by Dahlsgaard and Bodie [76]. Parents participated in seven sessions of training to effectively manage behaviors and expose their children to non-preferred foods. Picky eating and other problematic mealtime behaviors were evaluated by parents at pre-treatment, post-treatment, and in a three-month study follow-up. A significant reduction in picky eating behavior was observed post-treatment and was maintained at the follow-up. High parental adherence to and satisfaction with the training was also noted. This work indicates that strategies that combine parental and child intervention can also be useful in mitigating picky eating behavior [76].

## 5. Conclusions & Future Directions

The definition of and factors that lead to picky eating are multifaceted. Furthermore, the outcomes of picky eating and mealtime challenges can have a significant impact on families; thus, research studies to support healthy dietary intakes among young children are greatly needed. A strength of this review is its use of the categories of *nature* and *nurture* to illustrate the complexity of picky eating and its perception by parents and caregivers. On the other hand, limitations of this review include the potential for bias in study inclusion and that it was not conducted as an exhaustive review of the literature, as only one database was used with limited keywords and search terms.

Through this review process, we have identified several gaps in the literature that need to be addressed in order to capture the complexity of picky eating. Several different measurement tools, including a variety of questionnaires and surveys, are currently used to evaluate picky eating behavior leading to inconsistencies in study design, comparison, and interpretation of findings [5]. This area of work also relies largely on parental interpretation and reporting of picky eating behaviors which creates another source of variation [3]. Development of a consistent and objective approach to evaluate picky eating behavior, which includes an evidence-based definition that captures its multidimensionality, is essential.

There is also a lack of longitudinal research in this area. Adding to this research area with longitudinal data that utilizes a non-picky eater control will better inform parent and caregiver strategies to mitigate picky eating [5]. Longitudinal studies that investigate how the interaction across parents, children, and their environments affects eating behaviors are needed [41,64]. It is also important that future studies concentrate intervention strategies on the whole family rather than just on the child given the links between parent and child intake behaviors [7,76].

Cole et al. [19] established that there is an association between picky eating behavior and PROP taster genotype and suggest that PROP taster phenotype should be further explored. Furthermore, understanding the strategies that work best for children that exhibit food fussiness and picky eating behaviors along with strategies that are tailored to those with bitter sensitivity should be a focus of future studies [72,73]. Vegetables are the least liked food category amongst children, with dark green vegetables being the most disliked in this group [77,78]. Since they provide an excellent source of antioxidants, fiber, and carotenoids, and are important in reducing the risk of chronic diseases, children are a key target group for increasing their intake [72]. Identifying effective strategies for increasing vegetable intake during late infancy and early childhood is needed in order to establish long-term, healthful diets. Successful strategies will address the various influential factors that shape childhood food preferences and mealtime behaviors. As it is currently limited, research on the intersection of *nature* and *nurture* factors in relation to picky eating and difficult mealtime behaviors is also needed. This understanding will facilitate the development of innovative and personalized approaches to improving child feeding and nutrition.

## Figures and Tables

**Table 1:** Relationship between Parenting Styles and Perception of Child Feeding Behaviors, Parental Feeding Practices, & Consumption Patterns.

Parenting Style	Characterization [46]		Association with Parental Perception of Child Feeding Behavior [45]	Association with Parental Feeding Practices [50]	Association with Parental Food Practices [51]	Association with Child’s Consumption Patterns [52]
Permissive	Low control; High warmth	Lenient; Accepting of child impulses & actions	Positively associated with perception of picky eating behaviors	Negatively associated with modeling & monitoring	Negatively associated with mealtime structural practices, healthy food modeling, & household food rules	N/A
Authoritarian	High control; Low warmth	Exhibits control & regulation over child behaviors		Positively associated with restriction & pressure to eat; Negatively associated with monitoring	Negatively associated with mealtime structural practices & healthy food modeling	Negatively associated with vegetable consumption & availability of fruits and vegetables
Authoritative	High control; High warmth	Balance of control & child autonomy	Positively associated with non-picky eating behaviors	Negatively associated with restriction; Positively associated with modeling, monitoring, & perceptions of responsibility	Positively associated with mealtime structural practices & healthy food modeling	Positively associated with vegetable consumption & availability of fruits and vegetables

## References

[1] Jacobi C., Schmitz G., Stewart Agras W. (2008). Is Picky Eating an Eating Disorder?. Int. J. Eat. Disord..

[2] Samuel T.M., Musa-Veloso K., Ho M., Venditti C., Shahkhalili-Dulloo Y. (2018). A Narrative Review of Childhood Picky Eating and Its Relationship to Food Intakes, Nutritional Status, and Growth. Nutrients.

[3] Taylor C.M., Wernimont S.M., Northstone K., Emmett P.M. (2015). Picky/Fussy Eating in Children: Review of Definitions, Assessment, Prevalence and Dietary Intakes. Appetite.

[4] Birch L.L., Marlin D.W. (1982). I Don’t like It; I Never Tried It: Effects of Exposure on Two-Year-Old Children’s Food Preferences. Appetite.

[5] Taylor C.M., Emmett P.M. (2019). Picky Eating in Children: Causes and Consequences. Proc. Nutr. Soc..

[6] Walton K., Kuczynski L., Haycraft E., Breen A., Haines J. (2017). Time to Re-Think Picky Eating?: A Relational Approach to Understanding Picky Eating. Int. J. Behav. Nutr. Phys. Act..

[7] Johnson S.L. (2016). Developmental and Environmental Influences on Young Children’s Vegetable Preferences and Consumption. Adv. Nutr..

[8] Mikkilä V., Räsänen L., Raitakari O.T., Pietinen P., Viikari J. (2005). Consistent Dietary Patterns Identified from Childhood to Adulthood: The Cardiovascular Risk in Young Finns Study. Br. J. Nutr..

[9] Coulthard H., Blissett J. (2009). Fruit and Vegetable Consumption in Children and Their Mothers. Moderating Effects of Child Sensory Sensitivity. Appetite.

[10] Taylor C.M., Northstone K., Wernimont S.M., Emmett P.M. (2016). Macro-and Micronutrient Intakes in Picky Eaters: A Cause for Concern?. Am. J. Clin. Nutr..

[11] Dubois L., Farmer A., Girard M., Peterson K., Tatone-Tokuda F. (2007). Problem Eating Behaviors Related to Social Factors and Body Weight in Preschool Children: A Longitudinal Study. Int. J. Behav. Nutr. Phys. Act..

[12] Ekstein S., Laniado D., Glick B. (2010). Does Picky Eating Affect Weight-for-Length Measurements in Young Children?. Clin. Pediatr..

[13] Wright C.M., Parkinson K.N., Shipton D., Drewett R.F. (2007). How Do Toddler Eating Problems Relate to Their Eating Behavior, Food Preferences, and Growth?. Pediatrics.

[14] Carruth B.R., Skinner J.D. (2000). Revisiting the Picky Eater Phenomenon: Neophobic Behaviors of Young Children. J. Am. Coll. Nutr..

[15] Keller K.L., Steinmann L., Nurse R.J., Tepper B.J. (2002). Genetic Taste Sensitivity to 6-n-Propylthiouracil Influences Food Preference and Reported Intake in Preschool Children. Appetite.

[16] Tepper B.J., Nurse R.J. (1997). Fat Perception Is Related to PROP Taster Status. Physiol. Behav..

[17] Bell K.I., Tepper B.J. (2006). Short-Term Vegetable Intake by Young Children Classified by 6-n-Propylthoiuracil Bitter-Taste Phenotype. Am. J. Clin. Nutr..

[18] Mennella J., Nolden A., Bobowski N., Lumeng J., Fisher J. (2018). Measuring Sweet and Bitter Taste in Children: Individual Variation Due to Age and Taste Genetics. Pediatric Food Preferences and Eating Behaviors.

[19] Cole N.C., Wang A.A., Donovan S.M., Lee S.Y., Teran-Garcia M. (2017). Variants in Chemosensory Genes Are Associated with Picky Eating Behavior in Preschool-Age Children. J. Nutrigenet. Nutrigenom..

[20] Oftedal K.N., Tepper B.J. (2013). Influence of the PROP Bitter Taste Phenotype and Eating Attitudes on Energy Intake and Weight Status in Pre-Adolescents: A 6-Year Follow-up Study. Physiol. Behav..

[21] Mennella J.A., Bobowski N.K., Reed D.R. (2016). The Development of Sweet Taste: From Biology to Hedonics. Rev. Endocr. Metab. Disord..

[22] Mennella J.A., Pepino M.Y., Reed D.R. (2005). Genetic and Environmental Determinants of Bitter Perception and Sweet Preferences. Pediatrics.

[23] Petty S., Salame C., Mennella J.A., Pepino M.Y. (2020). Relationship between Sucrose Taste Detection Thresholds and Preferences in Children, Adolescents, and Adults. Nutrients.

[24] Fildes A., Van Jaarsveld C.H.M., Cooke L., Wardle J., Llewellyn C.H. (2016). Common Genetic Architecture Underlying Young Children’s Food Fussiness and Liking for Vegetables and Fruit. Am. J. Clin. Nutr..

[25] Cooke L.J., Haworth C.M., Wardle J. (2007). Genetic and Environmental Influences on Children’s Food Neophobia. Am. J. Clin. Nutr..

[26] Diószegi J., Llanaj E., Ádány R. (2019). Genetic Background of Taste Perception, Taste Preferences, and Its Nutritional Implications: A Systematic Review. Front. Genet..

[27] Berrichi M., Hichami A., Addou-Klouche L., Sayed Khan A., Khan N.A. (2020). CD36 and GPR120 Methylation Associates with Orosensory Detection Thresholds for Fat and Bitter in Algerian Young Obese Children. J. Clin. Med..

[28] Mennella J.A., Jagnow C.P., Beauchamp G.K. (2001). Prenatal and Postnatal Flavor Learning by Human Infants. Pediatrics.

[29] Beauchamp G.K., Mennella J.A. (2011). Flavor Perception in Human Infants: Development and Functional Significance. Digestion.

[30] Spahn J.M., Callahan E.H., Spill M.K., Wong Y.P., Benjamin-Neelon S.E., Birch L., Black M.M., Cook J.T., Faith M.S., Mennella J.A. (2019). Influence of Maternal Diet on Flavor Transfer to Amniotic Fluid and Breast Milk and Children’s Responses: A Systematic Review. Am. J. Clin. Nutr..

[31] Mennella J.A., Beauchamp G.K. (1993). Beer, Breast Feeding, and Folklore. Dev. Psychobiol..

[32] Mennella J.A., Beauchamp G.K. (1991). Maternal Diet Alters the Sensory Qualities of Human Milk and the Nursling’s Behavior. Pediatrics.

[33] Mennella J.A., Beauchamp G.K. (1992). The Transfer of Alcohol to Human Milk: Effects on Flavor and the Infant’s Behavior. Obstet. Gynecol. Surv..

[34] Mennella J.A., Beauchamp G.K. (1993). The Effects of Repeated Exposure to Garlic-Flavored Milk on the Nursling’s Behavior. Pediatr. Res..

[35] Mennella J.A., Beauchamp G.K. (1996). The Human Infants’ Response to Vanilla Flavors in Mother’s Milk and Formula. Infant Behav. Dev..

[36] Maier A.S., Chabanet C., Schaal B., Leathwood P.D., Issanchou S.N. (2008). Breastfeeding and Experience with Variety Early in Weaning Increase Infants’ Acceptance of New Foods for up to Two Months. Clin. Nutr..

[37] Mennella J.A., Nicklaus S., Jagolino A.L., Yourshaw L.M. (2008). Variety Is the Spice of Life: Strategies for Promoting Fruit and Vegetable Acceptance during Infancy. Physiol. Behav..

[38] Mennella J.A., Forestell C.A., Morgan L.K., Beauchamp G.K. (2009). Early Milk Feeding Influences Taste Acceptance and Liking during Infancy. Am. J. Clin. Nutr..

[39] Mennella J.A., Castor S.M. (2012). Sensitive Period in Flavor Learning: Effects of Duration of Exposure to Formula Flavors on Food Likes during Infancy. Clin. Nutr..

[40] Mennella J.A., Beauchamp G.K. (2002). Flavor Experiences during Formula Feeding Are Related to Preferences during Childhood. Early Hum. Dev..

[41] Blissett J., Fogel A. (2013). Intrinsic and Extrinsic Influences on Children’s Acceptance of New Foods. Physiol. Behav..

[42] Northstone K., Emmett P., Nethersole F. (2001). The Effect of Age of Introduction to Lumpy Solids on Foods Eaten and Reported Feeding Difficulties at 6 and 15 Months. J. Hum. Nutr. Diet..

[43] Coulthard H., Harris G., Emmett P. (2009). Delayed Introduction of Lumpy Foods to Children during the Complementary Feeding Period Affects Child’s Food Acceptance and Feeding at 7 Years of Age. Matern. Child Nutr..

[44] Greer F.R., Sicherer S.H., Wesley Burks A., Abrams S.A., Fuchs G.J., Kim J.H., Wesley Lindsey C., Magge S.N., Rome E.S., Schwarzenberg S.J. (2019). The Effects of Early Nutritional Interventions on the Development of Atopic Disease in Infants and Children: The Role of Maternal Dietary Restriction, Breastfeeding, Hydrolyzed Formulas, and Timing of Introduction of Allergenic Complementary Foods. Pediatrics.

[45] Podlesak A.K., Mozer M.E., Smith-Simpson S., Lee S.-Y., Donovan S.M. (2017). Associations between Parenting Style and Parent and Toddler Mealtime Behaviors. Curr. Dev. Nutr..

[46] Baumrind D. (1966). Effects of Authoritative Parental Control on Child Behavior. Child Dev..

[47] Boquin M., Smith-Simpson S., Donovan S.M., Lee S.Y. (2014). Mealtime Behaviors and Food Consumption of Perceived Picky and Nonpicky Eaters through Home Use Test. J. Food Sci..

[48] Robinson C., Mandleco B., Olsen S., Hart C., Touliatos J., Perlmutter B., Holden G. (2001). Parenting Styles and Dimensions Questionnaire (PSDQ). Handbook of Family Measurement Techniques: Instruments & Index.

[49] Cole N.C., An R., Lee S.Y., Donovan S.M. (2017). Correlates of Picky Eating and Food Neophobia in Young Children: A Systematic Review and Meta-Analysis. Nutr. Rev..

[50] Hubbs-Tait L., Kennedy T.S., Page M.C., Topham G.L., Harrist A.W. (2008). Parental Feeding Practices Predict Authoritative, Authoritarian, and Permissive Parenting Styles. J. Am. Diet. Assoc..

[51] Lopez N.V., Schembre S., Belcher B.R., O’Connor S., Maher J.P., Arbel R., Margolin G., Dunton G.F. (2018). Parenting Styles, Food-Related Parenting Practices, and Children’s Healthy Eating: A Meditation Analysis to Examine Relationships between Parenting and Child Diet. Appetite.

[52] Patrick H., Nicklas T.A., Hughes S.O., Morales M. (2005). The Benefits of Authoritative Feeding Style: Caregiver Feeding Styles and Children’s Food Consumption Patterns. Appetite.

[53] Goldman R.L., Radnitz C.L., McGrath R.E. (2012). The Role of Family Variables in Fruit and Vegetable Consumption in Preschool Children. J. Public health Res..

[54] Osborne C.L., Forestell C.A. (2012). Increasing Children’s Consumption of Fruit and Vegetables: Does the Type of Exposure Matter?. Physiol. Behav..

[55] Arcan C., Friend S., Flattum C.F., Story M., Fulkerson J.A. (2019). Fill “Half Your Child’s Plate with Fruits and Vegetables”: Correlations with Food-Related Practices and the Home Food Environment. Appetite.

[56] Rasmussen M., Krølner R., Klepp K.I., Lytle L., Brug J., Bere E., Due P. (2006). Determinants of Fruit and Vegetable Consumption among Children and Adolescents: A Review of the Literature. Part I: Quantitative Studies. Int. J. Behav. Nutr. Phys. Act..

[57] Harris H.A., Staton S., Morawska A., Gallegos D., Oakes C., Thorpe K. (2019). A Comparison of Maternal Feeding Responses to Child Fussy Eating in Low-Income Food Secure and Food Insecure Households. Appetite.

[58] Caton S.J., Ahern S.M., Remy E., Nicklaus S., Blundell P., Hetherington M.M. (2013). Repetition Counts: Repeated Exposure Increases Intake of a Novel Vegetable in UK Pre-School Children Compared to Flavour-Flavour and Flavour-Nutrient Learning. Br. J. Nutr..

[59] Forestell C.A., Mennella J.A. (2007). Early Determinants of Fruit and Vegetable Acceptance. Pediatrics.

[60] Birch L.L., McPhee L., Shoba B.C., Pirok E., Steinberg L. (1987). What Kind of Exposure Reduces Children’s Food Neophobia?. Looking vs. Tasting. Appetite.

[61] Mennella J.A., Reiter A.R., Daniels L.M. (2016). Vegetable and Fruit Acceptance during Infancy: Impact of Ontogeny, Genetics, and Early Experiences. Adv. Nutr..

[62] Coulthard H., Harris G., Emmett P. (2010). Long-Term Consequences of Early Fruit and Vegetable Feeding Practices in the United Kingdom. Public Health Nutr..

[63] Gerrish C.J., Mennella J.A. (2001). Flavor Variety Enhances Food Acceptance in Formula-Fed Infants. Am. J. Clin. Nutr..

[64] Wolstenholme H., Kelly C., Hennessy M., Heary C. (2020). Childhood Fussy/Picky Eating Behaviours: A Systematic Review and Synthesis of Qualitative Studies. Int. J. Behav. Nutr..

[65] Katherine Hoy M., Clemens J.C., Martin C.L., Moshfegh A.J. (2020). Fruit and Vegetable Consumption of US Adults by Level of Variety, What We Eat in America, NHANES 2013–2016. Curr. Dev. Nutr..

[66] Feng Y., Albiol Tapia M., Okada K., Castaneda Lazo N.B., Chapman-Novakofski K., Phillips C., Lee S.Y. (2018). Consumer Acceptance Comparison Between Seasoned and Unseasoned Vegetables. J. Food Sci..

[67] Manero J., Phillips C., Ellison B., Lee S.Y., Nickols-Richardson S.M., Chapman-Novakofski K.M. (2017). Influence of Seasoning on Vegetable Selection, Liking and Intent to Purchase. Appetite.

[68] Luu L., Manero J., Lee S.Y., Nickols-Richardson S.S., Chapman-Novakofski K. (2020). Role of Seasoning Vegetables on Consumer Behavior: Purchase, Intake, Liking, and Intention to Pay for Larger Servings. Food Qual. Prefer..

[69] Carney E.M., Stein W.M., Reigh N.A., Gater F.M., Bakke A.J., Hayes J.E., Keller K.L. (2018). Increasing Flavor Variety with Herbs and Spices Improves Relative Vegetable Intake in Children Who Are Propylthiouracil (PROP) Tasters Relative to Nontasters. Physiol. Behav..

[70] Savage J.S., Peterson J., Marini M., Bordi P.L., Birch L.L. (2013). The Addition of a Plain or Herb-Flavored Reduced-Fat Dip Is Associated with Improved Preschoolers’ Intake of Vegetables. J. Acad. Nutr. Diet..

[71] Fisher J.O., Mennella J.A., Hughes S.O., Liu Y., Mendoza P.M., Patrick H. (2012). Offering “Dip” Promotes Intake of a Moderately-Liked Raw Vegetable among Preschoolers with Genetic Sensitivity to Bitterness. J. Acad. Nutr. Diet..

[72] Keller K.L. (2014). The Use of Repeated Exposure and Associative Conditioning to Increase Vegetable Acceptance in Children: Explaining the Variability across Studies. J. Acad. Nutr. Diet..

[73] Nekitsing C., Blundell-Birtill P., Cockroft J.E., Hetherington M.M. (2018). Systematic Review and Meta-Analysis of Strategies to Increase Vegetable Consumption in Preschool Children Aged 2–5 Years. Appetite.

[74] Holley C.E., Haycraft E., Farrow C. (2015). “Why Don’t You Try It Again?” A Comparison of Parent Led, Home Based Interventions Aimed at Increasing Children’s Consumption of a Disliked Vegetable. Appetite.

[75] Musher-Eizenman D.R., Oehlhof M., Young K.M., Hauser J.C., Galliger C., Sommer A. (2011). Emerald Dragon Bites vs. Veggie Beans: Fun Food Names Increase Children’s Consumption of Novel Healthy Foods. J. Early Child. Res..

[76] Dahlsgaard K.K., Bodie J. (2019). The (Extremely) Picky Eaters Clinic: A Pilot Trial of a Seven-Session Group Behavioral Intervention for Parents of Children With Avoidant/Restrictive Food Intake Disorder. Cogn. Behav. Pract..

[77] Cooke L.J., Wardle J. (2005). Age and Gender Differences in Children’s Food Preferences. Br. J. Nutr..

[78] Fox M.K., Condon E., Briefel R.R., Reidy K.C., Deming D.M. (2010). Food Consumption Patterns of Young Preschoolers: Are They Starting Off on the Right Path?. J. Am. Diet. Assoc..

